# Pro-Inflammatory Oral Microbiota in Juvenile Spondyloarthritis: A Pilot Study

**DOI:** 10.3390/children9111764

**Published:** 2022-11-17

**Authors:** Matthew L Stoll, Jue Wang, Chung How Kau, Margaret Kathy Pierce, Casey D Morrow, Nicolaas C Geurs

**Affiliations:** 1 Department of Pediatrics, University of Alabama at Birmingham, Birmingham, AL 35233, USA; 2 Department of Pediatrics, Division of Pediatric Dentistry and Orthodontics, Cincinnati Children’s Hospital Medical Center, University of Cincinnati College of Medicine, Cincinnati, OH 45040, USA; 3 Department of Orthodontics, University of Alabama at Birmingham, Birmingham, AL 35294, USA; 4 Department of Cell, Developmental & Integrative Biology, University of Alabama at Birmingham, Birmingham, AL 35294, USA; 5 Department of Periodontology, University of Alabama at Birmingham, Birmingham, AL 35294, USA

**Keywords:** microbiota, gingiva, saliva, spondylarthritis

## Abstract

The role of the microbiota in the pathogenesis of arthritis is gaining increasing attention. While multiple studies have queried the intestinal microbiota, very few have analyzed the contents of the oral microbiota. In this pilot study, we obtained salivary and sub-gingival specimens from a cohort of six healthy controls and five children with well-controlled spondyloarthritis (SpA) and performed 16S sequencing on bacteria obtained from both habitats. The Quantitative Insight into Microbial Ecology tool suite was used to generate operational taxonomic units, Phyloseq was used for diversity analyses, and DeSeq2 was used to compare abundances while adjusting for multiple comparisons. A repeat specimen was obtained from one subject during a flare. Clustering based upon diagnosis was observed from both habitats, with decreased alpha diversity seen within the plaque obtained from the patients vs. controls. Among the differentially abundant taxa were statistically significantly increased plaque *Fusobacterium* and salivary *Rothia mucilaginosa* among the patients compared to the controls. Additionally, the abundance of plaque *Fusobacterium* increased in one patient at the time of a flare. Our data suggest that the oral cavity may harbor bacteria involved in the pathogenesis of spondyloarthritis; additional studies are warranted.

## 1. Introduction

The role of the microbiota in the pathogenesis of arthritis is gaining increasing attention. While there have been studies of the intestinal microbiota in patients with inflammatory bowel disease (IBD) since the 1950s [1], the last 10 years have witnessed an emerging interest of the microbiota in patients with multiple forms of arthritis, including spondyloarthritis (SpA) [2]. In this category of arthritis, which bears substantial clinical overlap with IBD, multiple studies have demonstrated altered fecal or mucosal bacterial populations, including increased abundance of *Ruminococcus* [3] and decreased abundance of *Faecalibacterium prausnitzii* [4], mirroring observations in subjects with IBD [5].

An understudied habitat in SpA is the oral cavity. One previous study involving adult patients with axial SpA did not demonstrate any differences in the contents of the microbiota as compared to healthy controls [6], although our previous work has shown that microbiota findings in adult subjects do not necessarily translate to pediatrics [7]. An altered sub-gingival microbiota was reported in pediatric patients with Crohn Disease [8]. Additionally, we have previously demonstrated that children with SpA have increased antibodies directed against an oral commensal organism [9].

To evaluate for a potential role of the oral microbiota in children with SpA, we performed a pilot study of the oral microbiota. Because different oral habitats have different associated bacteria in health [10], we studied two different habitats: the saliva and the sub-gingiva.

## 2. Patients and Methods

### 2.1. Overview

This was a cross-sectional study evaluating the contents of the salivary and sub-gingival microbiota of children with Juvenile SpA vs. healthy controls.

### 2.2. Subjects

Patients included in the study had either psoriatic juvenile idiopathic arthritis or enthesitis-related arthritis as per the International League of Associations for Rheumatology criteria for juvenile idiopathic arthritis (JIA) [11]. Controls were patients presenting for routine care at the Orthodontics clinic at the University of Alabama at Birmingham (UAB). Informed consent was obtained from the guardians of all subjects prior to performing any study-related procedures. The study was approved by the Institutional Review Board at UAB. We limited the study to children aged 10–18, to minimize variability introduced by primary vs. secondary dentition.

### 2.3. Collection of Samples

Collection of saliva samples was performed as previously described [10,12]. We collected 2–3 mL of unstimulated saliva into a 15 mL tube, centrifuged it at 3300× *g* for 10 min at 4 °C, and resuspended the pellet in Cary-Blair media [13]. This was kept frozen at −80 °C until use.

Collection of the sub-gingival samples was performed as previously described [10]. First, the supra-gingival plaque from multiple molars was removed with use of a Universal Columbia 13/14 Curette. Subsequently, the sub-gingival plaque was accessed and placed in a tube containing Cary-Blair media. As there does not appear to be much variation in the contents of the sub-gingival microbiota among healthy teeth [14], we pooled scrapings from multiple teeth to increase the yield. Screening for periodontitis was performed at the time of sample collection for the patients and at the onset of orthodontic treatment for the healthy controls.

### 2.4. Sequencing and Analysis of 16S Ribosomal DNA from the Salivary and Sub-Gingival Specimens

This was performed as previously described by our group [4,7,15]. Briefly, microbial genomic DNA was isolated using the Zymo mini kit (Irving, CA, USA), and the purified DNA was PCR-amplified at the Variable IV region from the 16S ribosomal DNA gene and sequenced on the MiSeq (Illumina, San Diego, CA, USA) device, generating 250 base-pair paired end reads. The initial analytic steps were performed with the Quantitative Insight Into Microbial Ecology (QIIME) tool suite [16] using open reference operational taxonomic unit (OTU) picking with uclust [17], with downstream steps performed with the R Phyloseq package [18]. Assessments of alpha diversity were performed with the chao1 test of richness and the Shannon measure of evenness. For assessment of beta diversity, we used the Permutational analysis of variance [19] test as administered by the Adonis test in the R package vegan [20] to the distance table generated with the Bray Curtis clustering algorithm to model whether the subject group predicted the structure of the microbiota. For assessment of taxonomic data, the major issue is controlling for multiple comparisons due to the large numbers of output data in the form of individual taxa or pathways, compounded by the non-normal distribution of the data and the large number of very low values (“excess zeros”). The Bioconductor package DeSeq2 [21], while designed for analysis of RNASeq data, can be used for microbiota data as well [22,23], as these issues are similar. It functions by normalizing the data and also removing from the analysis those output variables that are unlikely to confer meaningful differences between the groups due to near zero values, thus reducing the false discovery rate penalty [24], which was used. An FDR-corrected cutoff of 0.05 was considered statistically significant.

### 2.5. Power Calculations for a Definitive Study

As this was a pilot study, we performed power calculations for a hypothetical definitive study of the oral microbiota in children with SpA. To do so, we first performed community-wide power calculations using the R package HMP [25], which takes as input the OTU tables generated in the preliminary (in this case, current) study and estimates statistical power for various sample sizes. We also calculated statistical power for determining differences in individual taxa identified herein; this was performed with the R package RNASeqPower [26], which performs negative binomial model analysis for over-dispersed count data.

### 2.6. Statistical Analyses

Differences in alpha diversity were assessed using the Student’s *t*-test, using a significance threshold of 0.05. All analyses were performed with R version 4.2.0.

## 3. Results

### 3.1. Subjects

Eleven subjects aged 11–17 were included in the study. None of them had any remaining primary detention. Demographics as well as diagnosis and medication history (SpA subjects) are depicted in Table 1. No subjects were taking traditional disease-modifying anti-rheumatic drugs, although all five patients were on biologics for treatment of their arthritis. All subjects were in clinical remission at the time of the original assessment. One subject flared and had a repeat specimen obtained during the time of her flare. None of the subjects had evidence of periodontitis or gingivitis at the time of sample collection.

### 3.2. 16S Sequencing

Each subject underwent 16S sequencing of the sub-gingival as well as salivary microbiota. Read counts of approximately 50,000–60,000 were obtained from both groups and from both habitats (saliva and sub-gingiva), without significant intergroup differences (not shown).

**Figure 1:**
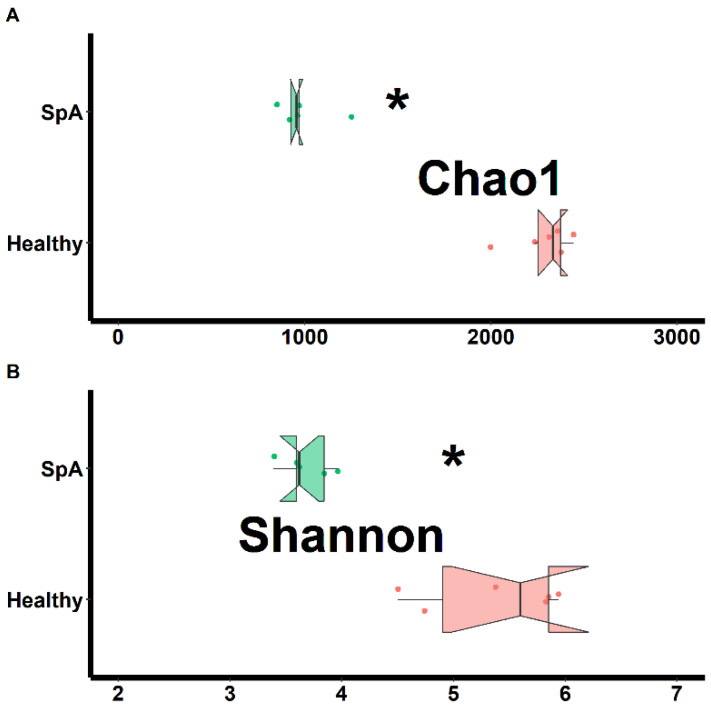
Assessment of richness (chao1; **A**) and evenness (Shannon; **B**) obtained from the sub-gingival sulcus of patients with Spondyloarthritis (SpA) and healthy controls. * indicates statistically significant at *p* < 0.01.

**Figure 2:**
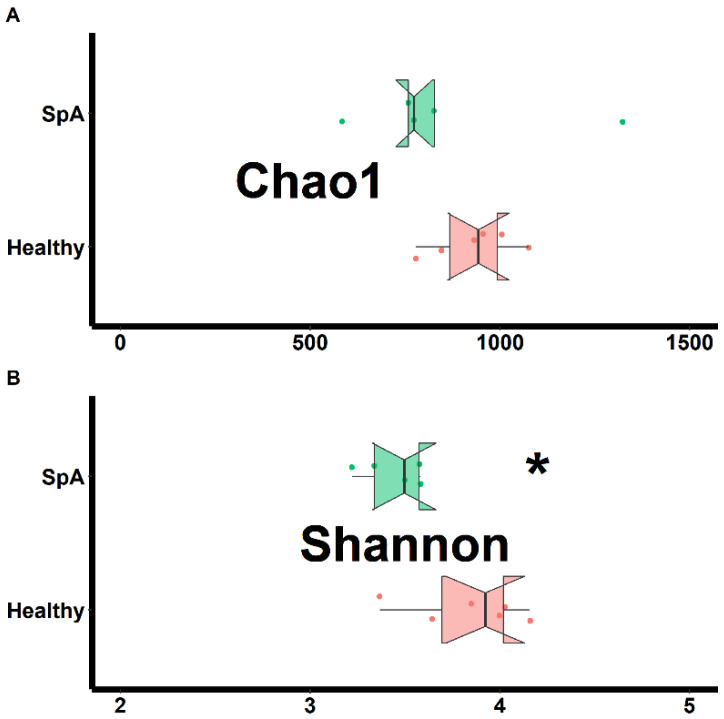
Assessment of richness (chao1; **A**) and evenness (Shannon; **B**) obtained from the saliva of patients with Spondyloarthritis (SpA) and healthy controls. * indicates statistically significant at *p* < 0.05.

Separate assessments of richness (chao1 test) and evenness (Shannon test) were performed on samples obtained from both habitats and shown in Figure 1 (sub-gingival plaque) and Figure 2 (saliva). Data from the plaque specimens showed significantly decreased richness (2287 ± 157 vs. 993 ± 154, *p* < 0.01) and evenness (5.4 ± 0.6 vs. 3.7 ± 0.2. *p* < 0.01) in the patient group, (Figure 1); potentially reflective of expansion of specific organisms within the SpA patients compared to controls. In contrast, the saliva specimens demonstrated similar richness (932 ± 107 vs. 853 ± 279, *p* = 0.578) and only marginally lower evenness (3.8 ± 0.3 vs. 3.4 ± 0.3, *p* = 0.020) among the patients vs. the controls, (Figure 2).

**Figure 3:**
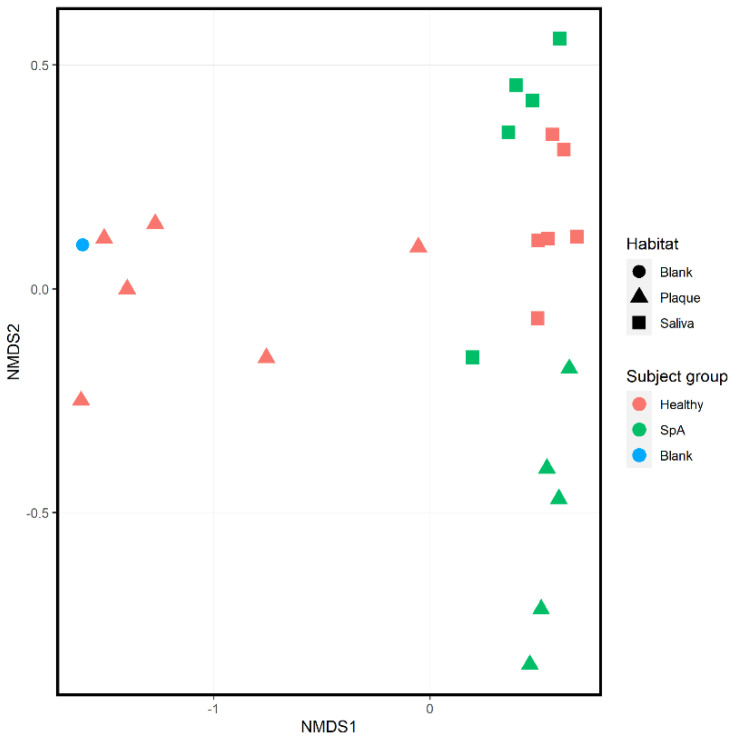
Ordination analysis of sub-gingival and salivary samples obtained from healthy control subjects and children with Spondyloarthritis. A negative control (blank) is included as well. The shape of the dot indicates the habitat (circle, blank specimen; triangle, plaque; square, saliva), while the color of the specimen indicates the subject group (blue, blank specimen; green, Spondyloarthritis; red, Healthy controls).

Ordination analysis performed on all 11 subjects inclusive of both habitats is shown in Figure 3; this includes a “blank” sample used as an environmental control. Regardless of patient group, there is apparent clustering based upon habitat (salivary samples depicted as squares, sub-gingival samples depicted as triangles), consistent with previously published data [10]. Additionally, within each habitat there is clustering based upon subject group, with the blank specimen clustering with most of the plaque specimens obtained from controls, consistent with the controls having low biomass in their sub-gingival sulci.

**Figure 4:**
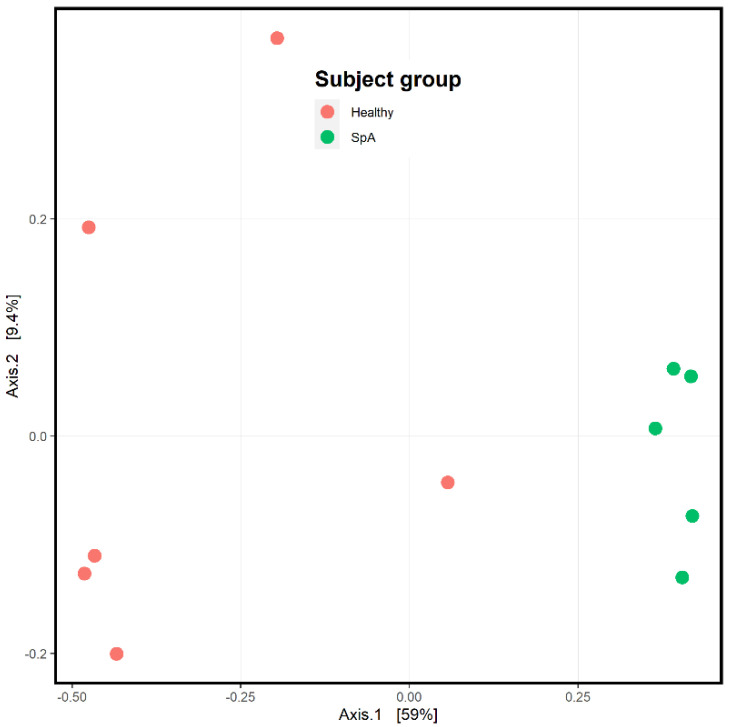
Ordination analysis of sub-gingival samples obtained from healthy control subjects and children with Spondyloarthritis (SpA). The SpA subjects are the green circles, while the controls are depicted in red.

**Figure 5:**
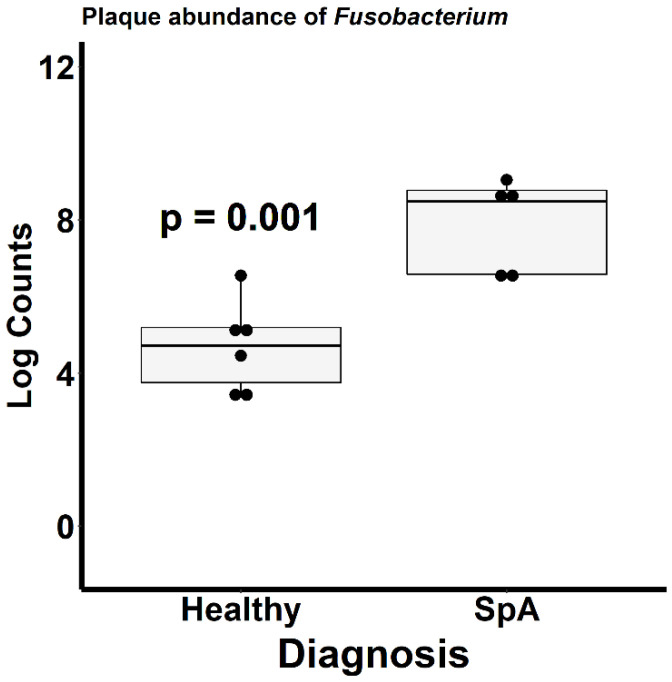
Relative abundance of *Fusobacterium* within the sub-gingival sulcus of patients with Spondyloarthritis (SpA) and healthy controls.

Figure 4 depicts the ordination analysis limited to the plaque specimens obtained from the patients and controls. Consistent with Figure 3, visually evident clustering between the two groups is shown, confirmed by the PermANOVA test (*p* = 0.004). To determine which organisms were driving this clustering, we used the DeSeq2 tool as described in the Methods section [21]. Even after correction for multiple comparisons, 1057 unique OTUs differentiated the two groups (Supplementary Table S1). Among the most abundant organisms was one matching to the *Fusobacterium* genus, which demonstrated a log2fold difference of 4.4 (*p* = 0.002; Figure 5), indicating significantly higher abundance in the patients. We obtained a repeat specimen on one subject six months after the initial assessment, at which time she was experiencing a flare manifested by clinical enthesitis and sacroiliitis; at this visit, her sub-gingival *Fusobacterium* abundance had increased from 8 to 16%.

**Figure 6:**
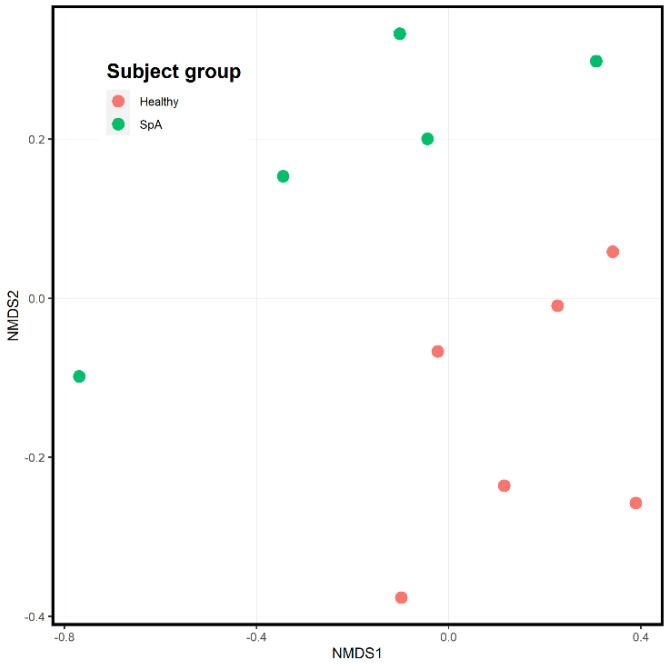
Ordination analysis of salivary samples obtained from healthy control subjects and children with Spondyloarthritis (SpA). The SpA subjects are the green circles, while the controls are depicted in red.

**Figure 7:**
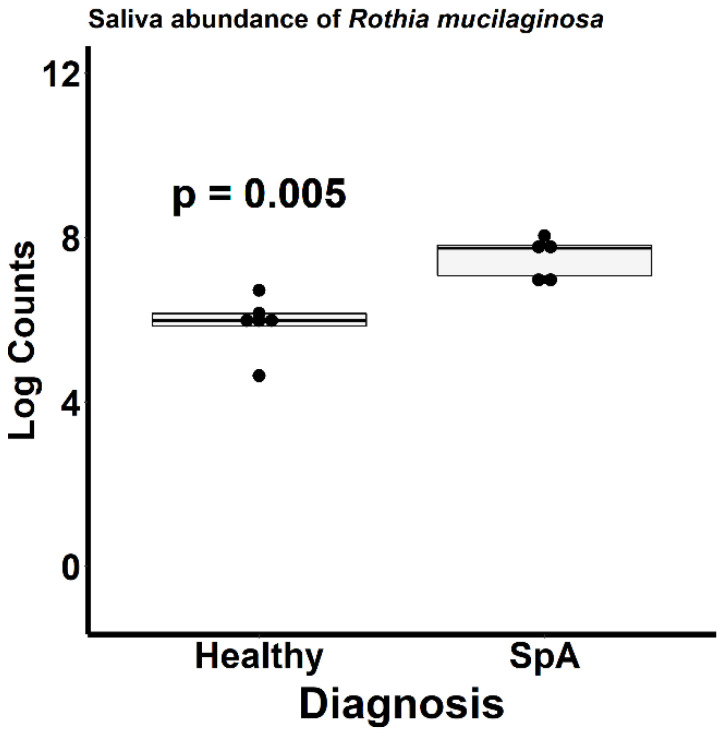
Relative abundance of *Rothia mucilaginosa* within the saliva of patients with Spondyloarthritis (SpA) and healthy controls.

Figure 6 depicts the ordination analysis limited to the saliva specimens obtained from the patients and controls. Here as well, visually evident clustering based upon diagnosis is present, confirmed by the PermANOVA test (*p* = 0.004). In contrast to the plaque specimens, where over 1000 OTUs were significantly altered between the patients and controls, DeSeq2 analysis identified only 34 OTUs differentiating the two groups (Table S2). Most of the organisms were enriched in the controls; however, the most abundant organism, *Rothia mucilaginosa*, was significantly more abundant in the patients (log2Fold change 2.2, *p* = 0.005; Figure 7).

### 3.3. Proposal for Definitive Study

We propose a study comparing the salivary and sub-gingival microbiota of treatment-naïve children with SpA and healthy controls, as one that would lay the groundwork for longitudinal studies that would assess whether changes in the microbiota could predict flare. The role of serologic reactivity against oral organisms should also be assessed [9]. The ultimate aim would be the evaluation of interventional efforts designed at improving periodontal health in children with SpA. As described in the Methods section, community-wide power calculations were performed with the R package HMP [25] using as input the OTU tables obtained from the salivary microbiota. We performed 1000 trials varying sample sizes from 20–30/group; finding that the projected power for these studies approaches 1 when sample size exceeds 20, indicating that relatively small sample sizes would still identify community-wide differences between patients and healthy controls. In light of the more pronounced changes in the sub-gingiva as compared to the saliva (e.g., Figure 3), even small sample sizes could theoretically identify community-wide differences in the sub-gingiva. Additionally, to determine sample sizes needed to identify statistically significantly differentially abundant pre-specified taxa (e.g., *Fusobacterium*), we used the RNASeqPower R Package [26], which is designed for these types of studies. Using the log2fold change obtained from the DeSeq2 calculations as the effect sizes, approximately 40 subjects would be required to identify differences in the salivary abundance of *Rothia mucilaginosa*, while a substantially lower number of subjects would be required to identify differences in sub-gingival *Fusobacterium*; larger sizes would be needed as the number of a priori comparisons increases.

## 4. Discussion

This pilot study demonstrated substantially altered sub-gingival microbiota between patients and controls, as well as altered salivary microbiota. The differentially abundant organisms identified herein are associated with dental caries. Specifically, among the patients, increased *Fusobacterium* was observed in the sub-gingival crevice, and increased *Rothia mucilaginosa* was observed in the saliva. *Fusobacterium nucleatum* is one of the organisms constituting the Red Complex bacteria, that have long been linked to periodontitis [27]. Likewise, salivary *Rothia mucilaginosa* has been linked to dental caries in children as well [28]. These data suggest a potential link between oral inflammation and SpA and are thus in agreement with our previously published work demonstrating that SpA patients had elevated IgA antibodies against a single oral commensal organism, *Prevotella oralis* [9]; albeit the latter was not identified as being differentially abundant in the current study. Further supporting a causal association between *Fusobacterium* and active disease is the finding that the abundance of this organism increased from 8 to 16% in a patient whose disease flared 6 months after the initial assessment. The mechanism by which oral inflammation may be linked to SpA is beyond the scope of the study. However, in light of findings showing involvement of the Th17 pathway in the setting of periodontitis [29,30] and findings from some [31,32,33] albeit not all [34] studies showing that patients with psoriasis or SpA had increased periodontal disease as compared to healthy controls, we suspect that local inflammation may generate an inflammatory response that extends systemically through cross-reactivity or other immunologic mechanisms. A recent review article has likewise reached the conclusion that periodontal inflammation can result in microbiota changes and SpA [35].

Of note, the findings reported herein appear to be specific to patients with juvenile SpA, compared to other categories of JIA. Specifically, studies of the sub-gingival and of the salivary microbiota in children with non-SpA types of JIA did not detect substantial differences in the composition of the microbiota between the patients and controls [36,37], underscoring potential pathophysiologic differences among the various forms of arthritis. 

This study has important limitations, particularly the small sample size. In addition, all of the patients were on immunomodulatory therapy. It is unknown whether these medications impact the oral microbiota, although the previous studies assessing the microbiota in children with JIA included patients on immunomodulatory therapy [36,37]. Additionally, similar to previously published studies in patients with IBD, JIA, or SpA, we did not perform detailed analyses of oral health or require specific oral hygiene practices prior to sample collection [6,8,36,37]. These findings will need to be validated with larger and optimally treatment-naïve cohorts. 

In summary, this pilot study suggests that children with SpA may have altered salivary and sub-gingival microbiota populations as compared to healthy children. The alterations may predispose to local inflammatory processes, potentially contributing to the onset and severity of the disease.

## Figures and Tables

**Table 1:** Demographic and clinical variables of the study population. Abbreviations: TNFi mAb, tumor necrosis factor inhibitor (monoclonal antibody).

Feature	Healthy Controls	Juvenile Spondyloarthritis
*n*	6	5
Demographics		
Male Sex	2 (33%)	1 (20%)
Race		
White	5 (83%)	5 (100%)
Black	1 (17%)	0
Age (years)	14.4 ± 2.1	14.3 ± 2.0
Treatment		
Etanercept	0	1 (20%)
TNFi mAb	0	3 (60%)
Abatacept	0	1 (20%)

## Data Availability

The sequence reads will be deposited into the Sequence Read Archive if the manuscript is accepted for publication.

## Supplementary Materials

The following supporting information can be downloaded at: https://www.mdpi.com/article/10.3390/children9111764/s1, Table S1. Statistically significant results of the DeSeq2 output of the sub-gingival plaque specimens comparing Juvenile Spondyloarthritis patients and healthy control subjects. Abbreviations: LFCSE, log2Fold Change standard error; padj, adjusted (corrected) *p*-value. LFC values > 0 represent operational taxonomic units higher in the SpA subjects. Table S2. Statistically significant results of the DeSeq2 output of the salivary specimens comparing Juvenile Spondyloarthritis patients and healthy control subjects. Abbreviations: LFCSE, log2Fold Change standard error; padj, adjusted (corrected) *p*-value. LFC values > 0 represent operational taxonomic units higher in the SpA subjects.
